# Fixing Data Gaps for Population Health in Africa: An Urgent Need

**DOI:** 10.3389/ijph.2022.1605418

**Published:** 2022-11-10

**Authors:** Yusuff Adebayo Adebisi, Don Eliseo Lucero-Prisno

**Affiliations:** ^1^ Faculty of Pharmacy, University of Ibadan, Ibadan, Nigeria; ^2^ Nuffield Department of Population Health, University of Oxford, Oxford, United Kingdom; ^3^ London School of Hygiene and Tropical Medicine, University of London, London, United Kingdom

**Keywords:** health data, population health, health intervention, Africa, health policy

African countries face a plethora of health concerns, and widespread data gaps have hampered the development of efficient and effective public health response plans. Making progress on population health efforts is essential in the transition to value-based, cost-effective, patient-centered, and quality care models, but it requires real-time, quality, and actionable data. The paucity of data can be a challenge when assessing the efficiency of various public health strategies. This is because the population health approach aims to improve health status and outcomes among a group of people rather than focusing on the health of one person at a time [1].

Most countries in Africa lack up-to-date, comprehensive, and accurate primary data on causes of death as well as health inequalities, making it difficult for them to track their progress toward the Sustainable Development Goals, evaluate the efficacy of programmatic interventions, establish health care priorities, and implement plans to improve health [2]. Lack of data also prevents most African governments from properly planning, monitoring, and evaluating public health projects. Poor data sourcing and classification, as well as human bias and mistakes, contribute to the inefficiencies of analogue data collection methods, which in turn lead to the implementation of crucial public health projects without appropriate data insights. As a result, many African countries have cemented a healthcare system that relies on estimates, is blind to crucial trends, and is ill-informed about the efficacy of policies that have already been put in place [3]. The demographic and health survey data that are common in many African countries are usually not large enough to produce reliable data of the levels or trends of some relatively rare health phenomena, annual data are usually not available and samples are generally not large enough to provide in-depth understanding into health issues in certain geographic areas.

The data gap in Africa is caused by a failure to capitalize on opportunities to collect useful population health data as well as lack of resources. For example, thousands of patients and caregivers visit health facilities every day, developing a strategy to collect data on public health issues both manually and electronically can be leveraged. Most health facilities lack a “health data department,” and even when one does exist, it serves only to record patient health data, not useful data that can be analyzed for population-based health interventions. The manual filing of patient health data across many facilities has made it more difficult to understand meaningful health trends at the population level. This made it critical for African countries to create a centralized health database system where useful data at the population level could be captured for health policies and interventions. Africa can learn from the UK Biobank model, which takes advantage of the National Health Service to collect an array of population health data [4]. It is time to move beyond single-centered health research in Africa, which is usually ineffective for health interventions, and start looking at health data holistically at the population level.

Antimicrobial resistance is a major global public health concern, and many African countries still lack population-level data on the burden, mortality, incidence, and prevalence of antimicrobial resistant infections, as well as the pattern of antibiotic consumption. This will, without a doubt, have an impact on response efforts and result in the waste of valuable resources. The first systematic analysis of the global burden of bacterial antimicrobial resistance published in the Lancet lends credence to the chronic severe data gap [5]. Creating a central database in African countries to capture health data in real time and manually across all levels of health care, public and private laboratories, and pharmacies will provide a factual understanding of the problem landscape and will help inform health policies. Climate change is also a threat to population health, and a lack of data makes it difficult to protect people from the effects of climate change on an especially vulnerable continent [6]. Due to severe data gaps, Africa has contributed the least to global warming and is also the least prepared to deal with the devastating consequences of rising temperatures [7]. Similarly, since the beginning of the COVID-19 pandemic, there have been significant data gaps and reporting inconsistencies regarding testing, case counts, and mortality statistics. These issues have all affected the capacity of African health authorities to adapt their responses to the scope and speed of the crisis and create the most efficient management strategies. It is hard to say enough about how important data is for making good health policies, especially at the population level.

There is an urgent need to invest in closing health data gaps, enhancing database systems and large-scale data collection processes and methods as well as data-related capacity building in Africa.
